# The Danger of Contagion in Throat Troubles

**Published:** 1885-10

**Authors:** 


### The Danger of Contagion in Throat Troubles.

The medical editor of *Babyhood* writes: A recent case within our knowledge, in which fatal diphtheria developed upon what had been believed to be a simple quinsy, suggests a few words regarding the duty of isolating any case of sore throat where there are other children in the house. Without entering upon any disputed points regarding diphtheria, it is generally agreed that the distinctive visible sign of it is its peculiar membranous deposit. A case may present clearly the conditions of a common sore throat," and subsequently diphtheria be unmistakably present. For our purpose it is unnecessary to discuss whether such cases are diphtheritic from the first or become so. The point for parents to know is that the sequence of dangerous symptoms upon those apparently slight is not uncommon, and that it is better for them to isolate a child fifty times unnecessarily than to be neglectful once.

We would urge, then, that, if at all possible, every child suffering from sore throat be isolated until it is distinctly convalescent. Physicians are often embarrassed, in urging the isolation of patients, by the timidity or suspiciousness of parents. If in such a case as has been described the physician recommends the precaution of isolation, the family, if of the timid type, is at once thrown into a panic, assuming that the physician really considers that the case is diphtheria or that he expects it will prove to be, and that he is concealing the facts, while really he is only taking proper sanitary precautions.

Other persons, on the other hand, immediately interpret the physician's frank statement of his reasons for isolating a supposed simple case as an evidence of want of knowledge on his part. They apparently think that to the properly educated physician diseases are as distinct and as easily discriminated as coins of different denominations. With such people the only course is to strongly advise isolation, and to give the reason for it, and to let them take the responsibility of neglecting the suggestion if they choose to do so. Intelligent people usually are grateful for the warning, even if it prove to have been unnecessary, and although they sometimes chaff the physician as "fussy."

